# The Effects of Aromatherapy Massage on Sleep Quality of Nurses on Monthly Rotating Night Shifts

**DOI:** 10.1155/2017/3861273

**Published:** 2017-07-06

**Authors:** Ying-Ying Chang, Chao-Ling Lin, Li-Yin Chang

**Affiliations:** ^1^Nursing Department, Taichung Veterans General Hospital, Taichung, Taiwan; ^2^St. Needs Aromatherapy Care Center, Palliative Ward, Taichung Veterans General Hospital, Taichung, Taiwan; ^3^Hungkuang University, Taichung, Taiwan

## Abstract

The goal of this study is to examine the effects of aromatherapy massage on sleep quality of nurses with monthly rotating night shifts. Subjects were enrolled at a medical center in central Taiwan with overall score ≥ 5 of Pittsburgh Sleep Quality Index (PSQI) and randomly assigned to the treatment or control groups. They were validated by pretests during their first graveyard shift in the trial period and the sleep quality information was collected by using the PSQI and sleep detectors. During the second graveyard shift, the treatment group received aromatherapy massage and the control group rested in the same aromatherapy room after work. All subjects filled out the PSQI surveys and the sleep quality information was collected during massage or resting and the following night. We found that the total PSQI was significantly decreased in the treatment group following the aromatherapy massage. Specifically, the components such as subjective sleep quality, sleep disturbance, and daytime dysfunction were significantly decreased. However, there were no significant changes of average PSQI scores between the two groups before and after intervention. Taken together, our study suggested that aromatherapy massage could improve sleep quality of nurses with monthly rotating night shift.

## 1. Introduction

Increasing evidence indicates that rotating night shifts not only affect health and work safety but also reduce the life quality of after-working hours. The main cause of that contradicts the circadian biological clock of humans [1, 2]. A stratified random sampling study presented that 67% of nurses were requested to work by rotating night shifts in national medical centers, regional hospitals, and local hospitals [3]. Furthermore, working in rotating night shifts also causes psychological stress leading to changes in lifestyle. The risks of chronic diseases including cardiovascular diseases are much higher in nurses with rotating night shifts than regular shifts [4]. Previously studies also indicated that nurses with rotating night shifts work have much higher inflammatory factors and risk of depression [5] and sleep disorders [6, 7]. Recent study pointed out that 50% of the nurses have sleep problems [8]. Hung and his colleagues found that nurses who worked on graveyard shifts had increased 2.26-fold of sleep disorders incidence compared to regular shifts [2]. A study also indicated that the turnover rate of nurses with night shift was 1.12-fold that of daytime shifts [9]. Thus, it is necessary to improve sleep problems of nurses who work with rotating shifts to prevent sleep disorders and increase overall life quality.

It has been shown that people with sleep disorders are suffering from fatigue and weakness of focus that often caused them to receive medications or nonmedicinal help [10]. Approximately 4.5% of American adults used some CAM (complementary and alternative medicine) to help them sleep, including body and mental relaxation therapies such as music therapy (39.1%) or bio/herbal therapies such as aromatherapy (64.8%) [11]. The word “aromatherapy” consists of “aroma” meaning a pleasant scent and “therapy” meaning healing. It incorporates the use of essential oils derived from fragrant plants which can be absorbed into the human body through airway or skin via massage [12]. Aromatherapy massage had been used as a therapeutic method to promote essential oil that could be absorbed by skin and then into the blood circulation through intensive touch with hands or massage tools. It has been reported that aromatherapy massage not only is able to benefit circulation, muscles, digestion, and lymphatic function, but also convey a feeling of being care, pampered, and valued to increase self-confidence [12, 13]. In fact, aromatherapy had been applied in several clinical practices. Cho et al. reported that patients who received aromatherapy had improved anxiety and sleep quality both before and after the surgery compared to nonreceiving patients [14]. Tang and Tse found that aromatherapy could decrease negative emotions in elders with chronic pain [15]. Chien et al. showed that aromatherapy significantly reduced PSQI scores in middle age career women [16]. In addition, aromatherapy also improved sleeping quality with decrease of PSQI scores in nurses who work with fixed night shifts, graveyard shifts, rotating triple shifts, or rotating shifts [17–19].

Although the NCCAM (National Center for Complementary and Alternative Medicine) Health Information suggests nonmedication alternative therapies are desirable strategies to improve sleep quality [11], the purpose of this study was to examine whether aromatherapy massage could improve sleep quality of nurses who work with monthly rotating night shifts.

## 2. Methods

### 2.1. Ethical Considerations

This study was reviewed and approved by the Investigational Review Board (IRB) (TCVGH-1037419D) and nurses on monthly rotating shifts were recruited from a medical center in central Taiwan. Subjects who met the inclusion criteria and signed informed consent after the principal investigator explained the study purpose and processes were enrolled.

### 2.2. Participants

The inclusion criteria were (1) female nurses on monthly rotating shifts, (2) having a total PSQI ≥ 5, and (3) age between 20 and 50 years. The exclusion criteria were (1) working fixed night or day shifts, (2) pregnancy, and (3) menopause.

### 2.3. Experimental Design

This study adopted a randomized controlled trial design. Randomization was assigned by a computer and information regarding group assignment was sealed in envelopes. Participants received the envelopes revealing the study group assignment.

The pretest data of PSQI score and sleep quality were collected in both groups during the first graveyard shift. All subjects finished the PSQI survey of previous week and then laid down for one hour with soothing background music and simultaneously collected their sleep quality information by using a take-home sleep detector. All subjects then returned home and continued to collect their sleep information at the same day.

**Figure 1:**
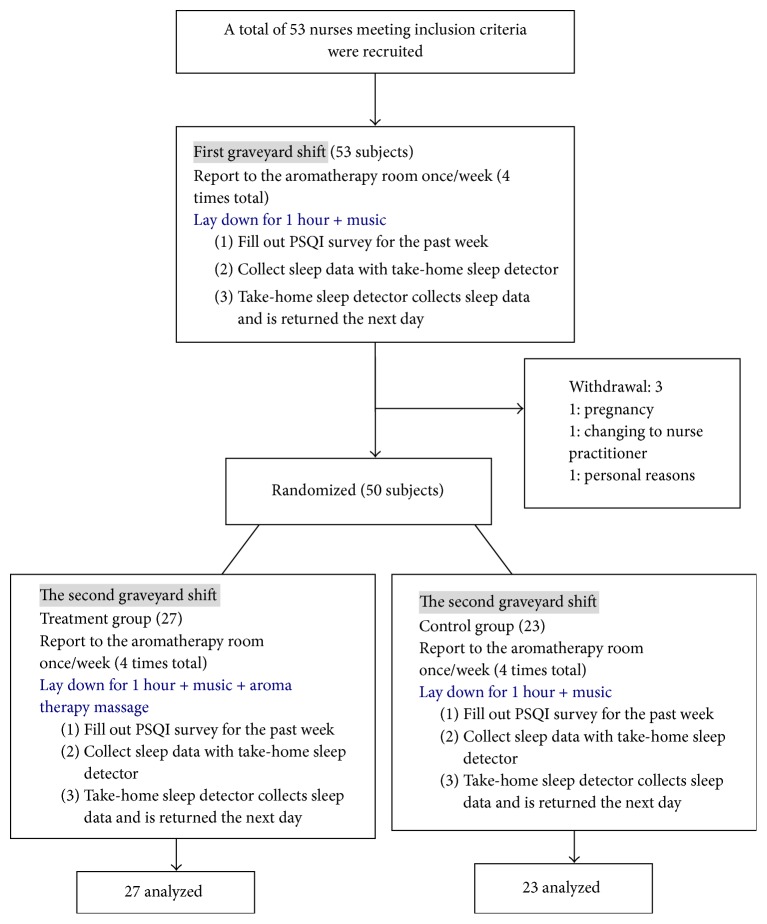
Study process.

After a week of the pretests, the data was collected for the following four weeks of the second graveyard shift. All subjects filled out a PSQI survey for the previous week and received an aroma therapy massage or laid down for rest in the same aromatherapy room with the same soothing background music. The sleep quality information was also collected during the interventions and night's sleep at the same day by using the same sleep detector (the schematic protocol of this study is shown in Figure 1).

### 2.4. Intervention: Aromatherapy Massage

The participants removed all accessories and electronics and then relaxed and laid down at the aromatherapy room for 5 minutes with the same slow music as background. The following aromatherapy massage was processed by the same aromatherapist with IFPA (the International Federation of Professional Aromatherapists) British international professional aromatherapist league license. The aromatherapist used the essential oil dripping into the heart Chakra (chest) to allow the subject to sense its scent and then gently rocked the body with essential oil and massage oil of the subject to promote relaxation of body muscle for the following 25 minutes. The body regions including head, shoulders, and neck were massaged to promote absorption of the essential oils and help them relax. The participants then drank 300 cc of warm water and rested for 30 minutes.

The materials of essential oil and massage oil are presented as follows.

*(1) Essential Oils*. The main chemical component of the essential oil is Terpinen-4-ol (36%) which is steam extracted from sweet marjoram (scientific name is* Origanum majorana* sweet, a member of Lamiaceae family) [20, 21].

*(2) Massage Oil Formula*. 5 ml of sweet almond oil is combined with 100 *μ*l of sweet marjoram essential oil (2%) and then gently mixed at room temperature until it is homogeneously liquid.

### 2.5. Measurements

(1) Pittsburgh Sleep Quality Index (PSQI) was developed by Buysse et al. [22]. Its 7 major components include subjective sleep quality, sleep latency, sleep duration, habitual sleep efficiency, sleep disturbance, daytime dysfunction, and use of sleeping medication. The score for each component ranges from 0 to 3; higher scores represent poorer sleep quality. Total score ranges from 0 to 21 and a total score > 5 indicates poor sleep quality [23]. The Cronbach alpha coefficient for PSQI internal consistency reliability was 0.83, and after two weeks, the test/retest reliability correlation coefficient was* R* = 0.85–0.90. (2) Take-home sleep detector, Ezsleep (TX-EK3), is an electrocardiogram (ECG) signal collector (see Figure 1). Its ECG signal detection method is owned by the American company DynaDx and its system requirements have also passed DynaDX validation. It has received US FDA510(k) clearance (K070855) and Taiwan TFDA medical device clearance (021186). The safety standards comply with medical electrical equipment technical standards IEC6060-1 and IEC61000. This monitor provides analysis such as duration of onset, duration of deep sleep and ratio, duration of light sleep and ratio, duration of wake/dream time and ratio, and apnea-hypopnea index (AHI). AHI represents the average number of apnea events per hour; each event is defined by a pause or decrease (less than 50% of normal) in breathing during sleep for a period of more than 10 seconds: normal: <5/hour; mild: 5–15/hour; moderate: 15–30/hour; and severe: >30/hour. According to the healthy sleep standards proposed by Harvard University Medical School, onset should occur within 30 minutes; the ratio of deep sleep should be higher than 41.1%, light sleep ratio less than 36.4%, dream/wake ratio less than 21.6%, and AHI less than 5/hour. Subjects also self-reported the beginning and end time of sleep, subjective sleep quality, and whether the total waking time after initially falling asleep exceeded 30 minutes. The ranking of subjective sleep quality was very good (5), good (4), fair (3), poor (2), and very poor (1); subjects chose the rank that best represented their sleep quality throughout the previous night.

### 2.6. Data Analysis

Statistical analysis of the study data was conducted using the SPSS 18.00 software suite. The *t*-test, Chi-square test, Fisher's exact test, paired *t*-test, and Wilcox signed rank test were used to compare the group differences for each variable; generalized estimating equations (GEE) were used to determine the effects of the treatment.

## 3. Results

The study recruited 53 subjects initially. Three subjects withdrew from the study during the first 4 weeks of data collection in the initial graveyard shift; one withdrew due to pregnancy, one transferred to a new position as a nurse practitioner with a normal work schedule, and one withdrew due to personal career planning. At study termination, complete experimental data were successfully collected from 50 subjects, with 27 randomized to the treatment group and 23 to the control group. The average age of the subjects was 29.37 ± 5.37 (range 23–48) and the average number of years working at the study site was 5.62 ± 4.68 (range 1.73–32.9). The two groups had no significant difference in demographics such as age, number of years working at the study site, BMI, job title, career status, advanced skills, and educational background (Table 1).

The analysis of PSQI is summarized in Table 2. The treatment group had a significant decrease (i.e., better sleep) in PSQI after aromatherapy as compared to before (*Z* = −3.54,* P* < 0.001). Specifically, there was a significant decrease in subjective sleep quality (*Z* = −3.38,* P* = 0.001), sleep disturbance (*Z* = −2.99,* P* = 0.003), and daytime dysfunction (*Z* = −3.57,* P* < 0.001). On the other hand, the control group had no significant difference in the total PSQI, but a significant decrease in daytime dysfunction (*Z* = −3.08,* P* = 0.002). The difference between the two groups in total PSQI scores was analyzed with GEE (Table 3). The PSQI of the treatment group was not different from the control group prior to pretesting (*P* = 0.64). Thus, there was no testing effect between the two groups. There was a significant difference in the number of repeated tests (*P* = 0.000). Additionally, as compared to the pretest, the control group started showing significant differences from the second test (*P* = 0.003) during pretesting, indicating that the control group also had a gradually decreasing PSQI. After taking the testing effect and the growth effect of the control group into consideration, there was no significant difference (*P* = 0.565) between the treatment group and the control group in average change in PSQI after intervention.

In take-home sleep detector measurements as shown in Table 4, the treatment group demonstrated a significant increase in subjective sleep quality score (*Z* = −2.62,* P* = 0.009) (i.e., better subjective sleep quality) while in the aromatherapy room, as well as a significant decrease in sleep duration (hours) (*Z* = −2.22,* P* = 0.026). On the other hand, no difference was observed in sleep onset, deep sleep, light sleep, wake/dream time, and AHI in control group. For the take-home sleep detector measurements taken at home or at dorm that day, neither the treatment group nor the control group demonstrated a significant difference. GEE analysis showed a significant (*P* = 0.011) improvement in subjective sleep quality in the treatment group as compared with control group (Table 5). No significant changes were observed in sleep onset time, deep sleep, light sleep, wake/dream time, and AHI between the two groups, whether in the aromatherapy room or at home/dorm.

## 4. Discussions

The average sleep time of nurses in this study (5.80−5.90 hours) was less than the 6.5−8.5 hours needed for adults [24]. In this study, among 450 PSQI surveys (50 subjects, 9 surveys each), 19 (4.2%) indicated the use of sleep medications. This number aligns with findings from several sleep quality studies performed on nurses [2, 17, 25].

In the medical field, a common consensus is that medication is not the best alternative for insomnia. Buysse proposes using behavioral and cognitive therapies as priority choices to deal with sleep problems [26]. The NCCAM Health Information suggests nonmedication alternative therapies to improve sleep quality [11]. Reduction of PSQI in the treatment group of this study was similar with previous studies of aromatherapy using lavender oil. In the following studies, massage was not implemented. In Yangs study, two drops of lavender oil were applied to the pillow for sniffing, every day before sleep for 12 weeks [17]. In Chien et al.'s study, participants inhaled lavender oil for 20 min each time [16]. The experiment was conducted twice per week for 12 weeks. Another aromatherapy experiment conducted by Hsi et al. used formulated massage oil with essential lavender oils, grapefruit extract, clary sage, and neroli. The essential oil was applied to the participants skin to improve sleep quality [19].

However, there are some aromatherapy studies that do not show significant differences in improving sleep quality. Lewith et al. conducted an experiment with 18−50-year-old group with moderate sleep disorders. Subjects were randomly assigned into two groups. Participants used alternately sweet almond oil and lavender essential oil intervention steam inhalation at the home. Steam inhalation of lavender essential oil improved PSQI scores, but the two kinds of interventions indicated no significant difference (*P* = 0.07) on improving sleep quality [27]. Hirokawa and his colleagues recruited the students without insomnia or sleep disorders. Participants were given lavender essential oil. They opened the bottle and let the natural volatile in the air and sniffed at bedtime. This intervention helped the participants fall asleep (*P* = 0.01) but did not extend sleep time (*P* = 0.93) [28].

In Ju et al.'s study massage was implemented with formulated oil. The massage oil was blended essential oils of lavender, marjoram, ylang ylang, and neroli in a 20 : 10 : 15 : 2 ratio with a carrier oil base of almond and jojoba oils in a 9 : 1 ratio. The formulated massage oil was used to perform aroma therapy massage on middle age women with hypertension. The treatment group had a significant reduction in both systolic and diastolic blood pressure and also a significant improvement in sleep quality (Tukey,* P* < 0.05) [29]. The results prove that the essential oil used in this study on the autonomic nervous system to increase the quality of sleep was effective.

Aside from the commonly used lavender, sweet marjoram essential oil can be further studied for its relaxing and sleep-aiding effects. Sweet marjoram contains several compounds which aid sleep. These include linalyl acetate which calms the emotion, linalool which stabilizes the cardiovascular system and relaxes the body, monoterpenes (such as a-pinene, y-pinene, and p-cymene) which have anti-inflammatory and mild antibacterial properties, and terpinen-4-ol which helps dilate blood vessels and regulate the nervous system [20, 29]. Ono (2000) pointed out that lavender, Roman chamomile, neroli, orange, balloon flower, clary sage, and marjoram oils are used to help sleep because of their effects on muscle relaxation and emotional calming. A more stringent study design is required to validate whether this is an effect of the sweet marjoram [20].

This study employed the same sleep environment setup and soothing music for both groups, which may explain why there was no significant difference in the changes in pre- and posttest results between the two groups. It is not clear whether the implementation of music in both groups affects the results of sleep quality.

Lin et al. studied nurses working at intensive care units (ICU) in hospitals with different methods of shift rotation, which included 30 nurses on fixed shifts and 30 on irregular shifts. Both groups were evaluated for three consecutive days using PSQI and take-home sleep detectors. No group difference was found with regard to poor sleep quality and sleep quality measured by the take-home detector [30]. A significant difference was found (*P* = 0.011) in the changes in subjective sleep quality between the two groups when comparing the results of pretests and posttests conducted in the aromatherapy room. However, there was no significant difference in the changes in sleep quality measurements either in aromatherapy room or at home/dorm. Further studies may be required to validate the credibility of sleep quality measurements taken from the sleep-sensor apparatus used in this study.

In conclusion, our study found that aroma therapy massage is an effective treatment in improving sleep quality (PSQI score and subjective sleep quality) of the nurses on monthly rotating night shifts. Establishment of aroma therapy massage as a part of employee health improvement programs especially for those required to work night shifts is recommended as an option to alleviate fatigue and promote sleep. Due to limitations such as human resources, materials, time, and work schedule, it was not possible to collect a large sample number for this study. Future investigations can focus on the use of different essential oils, their methods of application, dosage, demographics, and so on to conduct broader and more thorough research and to confirm differences in their therapeutic effects and their value in promoting health.

## Figures and Tables

**Table 1:** Comparison of demographics between the two groups.

Variable	Treatment (*n* = 27)				Control (*n* = 23)				*t* or *χ*^2^	*P*
	*n*	%	Mean	SD	*n*	%	Mean	SD		
Age	27		28.36	4.67	23		30.55	5.98	−1.45	0.153
Number of years at the hospital	27		4.67	4.09	23		6.74	5.17	−1.55	0.129
BMI	27		22.76	4.17	23		21.71	3.09	1.00	0.324
Job title										
Contract nurse	18	58.10			13	41.90			0.54	0.461
Government employee	9	47.40			10	52.60				
Skill advancement									1.83^a^	0.672
N	6	75.00			2	25.00				
N1	7	53.80			6	46.20				
N2	10	47.60			11	52.40				
≧N3	4	50.00			4	50.00				
Highest degree									2.34^a^	0.451
Associate	0	0.00			2	100.00				
Bachelor	26	56.50			20	43.50				
Master	1	50.00			1	50.00				

^a^Fisher's exact test.

**Table 2:** Analysis of differences in PSQI between the two groups before and after intervention.

Variable	Treatment (*n* = 27)						Control (*n* = 23)					
	Pretest		Posttest		*Z*^a^	*P*	Pretest		Posttest		*Z*^a^	*P*
	Mean	SD	Mean	SD			Mean	SD	Mean	SD		
Subjective sleep quality	1.65	0.42	1.32	0.35	−3.38	0.001^*∗∗∗*^	1.38	0.38	1.27	0.39	−0.91	0.360
Sleep latency	1.49	0.81	1.27	0.64	−1.91	0.056	1.29	0.87	1.30	0.69	−0.29	0.771
Sleep duration	1.53	0.75	1.35	0.58	−1.47	0.140	1.22	0.79	1.08	0.56	−0.70	0.484
Habitual sleep efficiency	1.02	0.65	1.12	0.78	−0.75	0.453	1.14	0.93	1.38	0.89	−0.96	0.339
Sleep disturbance	1.18	0.30	1.01	0.21	−2.99	0.003^*∗∗*^	1.12	0.34	1.02	0.35	−1.12	0.265
Use of sleeping medication	0.11	0.53	0.14	0.50	−0.82	0.414	0.02	0.10	0.01	0.05	−0.45	0.655
Daytime dysfunction	1.92	0.48	1.42	0.55	−3.57	0.000^*∗∗∗*^	1.90	0.73	1.51	0.79	−3.08	0.002^*∗∗*^
PSQI total score	8.89	2.45	7.63	2.22	−3.54	0.000^*∗∗∗*^	8.08	2.31	7.58	1.92	−1.31	0.191

^a^Wilcox signed rank test; ^*∗∗*^*P* < 0.01 and ^*∗∗∗*^*P* < 0.001.

**Table 3:** PSQI repeated test GEE analysis (*N* = 50).

Parameter	Estimated B value	Standard error	95% Wald confidence interval		Wald Chi-square	*P* value
			Upper limit	Lower limit		
Intercept	9.52	.63	8.29	10.76	228.67	0.000
Treatment group	.40	.87	−1.29	2.10	.22	0.641
Control group	0	.	.	.	.	.
4th posttest	−2.17	.56	−3.26	−1.08	15.31	0.000
3rd posttest	−2.13	.61	−3.33	−.93	12.09	0.001
2nd posttest	−1.30	.61	−2.51	−.10	4.48	0.034
1st posttest	−2.17	.59	−3.33	−1.01	13.49	0.000
4th pretest	−1.74	.57	−2.85	−.63	9.39	0.002
3rd pretest	−1.78	.43	−2.63	−.93	16.82	0.000
2nd pretest	−1.39	.47	−2.31	−.47	8.72	0.003
1st pretest	−.87	.47	−1.80	.06	3.37	0.066
Prior to pretest	0	.	.	.	.	.
[Treatment Group]*∗*[4th Posttest]	−.53	.92	−2.33	1.273	.33	0.565
[Treatment Group]*∗*[3rd Posttest]	−.76	.87	−2.45	.938	.77	0.381
[Treatment Group]*∗*[2nd Posttest]	−.55	.81	−2.14	1.043	.45	0.500
[Treatment Group]*∗*[1st Posttest]	.43	.76	−1.07	1.932	.32	0.571
[Treatment Group]*∗*[4th Pretest]	.44	.78	−1.08	1.970	.32	0.570
[Treatment Group]*∗*[3rd Pretest]	.52	.71	−.86	1.906	.55	0.458
[Treatment Group]*∗*[2nd Pretest]	.50	.86	−1.19	2.193	.34	0.560
[Treatment Group]*∗*[1st Pretest]	.17	.68	−1.17	1.507	.06	0.808
[Treatment Group]*∗*[Prior to Pretest]	0	.	.	.	.	.
[Control Group]*∗*[4th Posttest]	0	.	.	.	.	.
[Control Group]*∗*[3rd Posttest]	0	.	.	.	.	.
[Control Group]*∗*[2nd Posttest]	0	.	.	.	.	.
[Control Group]*∗*[1st Posttest]	0	.	.	.	.	.
[Control Group]*∗*[4th Pretest]	0	.	.	.	.	.
[Control Group]*∗*[3rd Pretest]	0	.	.	.	.	.
[Control Group]*∗*[2nd Pretest]	0	.	.	.	.	.
[Control Group]*∗*[1st Pretest]	0	.	.	.	.	.
[Control Group]*∗*[Prior to Pretest]	0	.	.	.	.	.

**Table 4:** Analysis of take-home sleep detector between the two groups before and after intervention.

Variable	Treatment group (*n* = 26)						Control group (*n* = 23)					
	Pretest		Posttest		*Z* ^a^	*P*	Pretest		Posttest		*Z* ^a^	*P*
	Mean	SD	Mean	SD			Mean	SD	Mean	SD		
Aromatherapy room												
Subjective sleep quality	2.99	0.75	3.57	0.63	− 2.62	.009^*∗∗*^	2.97	0.72	2.97	0.43	−0.05	0.959
Sleep duration (hr)	1.17	0.32	1.09	0.07	−0.79	.427	1.29	0.65	1.15	0.27	−0.49	0.625
Onset time (minutes)	13.98	6.09	18.23	9.60	−1.72	.086	11.33	5.64	12.08	6.59	−0.84	0.401
Deep sleep duration (hr)	0.72	0.22	0.59	0.26	−2.22	.026^*∗*^	0.72	0.42	0.68	0.33	−0.09	0.927
Light sleep duration (hr)	0.21	0.19	0.25	0.17	−1.01	.312	0.31	0.22	0.27	0.20	−1.13	0.257
Total wake/dream time (hr)	0.19	0.14	0.23	0.14	−1.92	.055	0.24	0.25	0.18	0.11	−0.23	0.821
AHI (count)	2.48	7.65	2.15	4.33	−0.24	.807	5.23	9.55	6.74	11.38	−0.72	0.469
Home or dorm												
Subjective sleep quality	3.35	0.70	3.41	0.83	−0.37	.715	3.31	0.77	3.39	0.90	−0.26	0.795
Sleep duration (hr)	5.96	2.24	5.93	1.53	−0.14	.889	5.80	1.67	5.98	1.59	−0.63	0.527
Onset time (minutes)	27.00	32.41	28.29	28.00	−0.15	.882	23.89	14.92	30.45	22.60	−0.83	0.408
Deep sleep duration (hr)	2.57	1.37	2.55	1.03	−0.13	.899	2.27	1.09	2.35	0.90	−0.81	0.417
Light sleep duration (hr)	1.75	1.03	1.71	0.74	−0.61	.542	1.94	0.65	1.91	0.74	−0.24	0.808
Total dream time (hr)	1.50	0.76	1.51	0.54	−0.20	.839	1.46	0.47	1.57	0.54	−1.01	0.313
AHI (count)	3.72	4.86	3.63	4.01	−0.34	.737	5.29	5.68	5.24	4.96	−0.50	0.614

^a^Wilcox signed rank test; ^*∗*^*P* < 0.05 and ^*∗∗*^*P* < 0.01.

**Table 5:** GEE analysis of the two groups' repeated measurement with subjective sleep quality at aromatherapy room.

Parameter	Estimated value of B	Standard error	95% Wald confidence interval		Wald Chi-square	*P* value
			Lower limit	Upper limit		
Intercept	2.97	.15	2.68	3.26	404.76	0.000
Treatment group	.02	.21	−.39	.42	.01	0.925
Control group	0	.	.	.	.	.
Posttest	−.004	.13	−.26	.26	.001	0.978
Pretest	0	.	.	.	.	.
[TreatmentGroup]*∗*[Posttest]	.58	.23	.14	1.03	6.54	0.011
[Treatment Group]*∗*[Pretest]	0					
[Control Group]*∗*[Posttest]	0	.	.	.	.	.
[Control Group]*∗*[Pretest]	0	.	.	.	.	.
(Scale)	.42					
